# The Little Ice Age signature in a 700-year high-resolution chironomid record of summer temperatures in the Central Eastern Alps

**DOI:** 10.1007/s00382-018-4555-y

**Published:** 2018-12-01

**Authors:** Elena A. Ilyashuk, Oliver Heiri, Boris P. Ilyashuk, Karin A. Koinig, Roland Psenner

**Affiliations:** 1 Institute of Ecology, University of Innsbruck, Technikerstraße 25, 6020 Innsbruck, Austria; 2 Geoecology, Department of Environmental Sciences, University of Basel, Klingelbergstrasse 27, 4056 Basel, Switzerland; 3 Institute for Alpine Environment, Eurac Research, Viale Druso 1, 39100 Bozen/Bolzano, Italy

**Keywords:** Alps, Chironomidae, Little Ice Age, LTER, Mountain lake, Paleotemperature

## Abstract

Despite the fact that the Little Ice Age (LIA) is well documented for the European Alps, substantial uncertainties concerning the regional spatio-temporal patterns of temperature changes associated with the LIA still exist, especially for their eastern sector. Here we present a high-resolution (4–10 years) 700-year long mean July air temperature reconstruction based on subfossil chironomid assemblages from a remote lake in the Austrian Eastern Alps to gain further insights into the LIA climatic deterioration in the region. The record provides evidence for a prolonged period of predominantly cooler conditions during AD 1530–1920, broadly equivalent to the climatically defined LIA in Europe. The main LIA phase appears to have consisted of two cold time intervals divided by slightly warmer episodes in the second half of the 1600s. The most severe cooling occurred during the eighteenth century. The LIA temperature minimum about 1.5 °C below the long-term mean recorded in the mid-1780 s coincides with the strongest volcanic signal found in the Greenland ice cores over the past 700 years and may be, at least in part, a manifestation of cooling that followed the long-lasting AD 1783–1784 Laki eruption. A continuous warming trend is evident since ca AD 1890 (1.1 °C in 120 years). The chironomid-inferred temperatures show a clear correlation with the instrumental data and reveal a close agreement with paleotemperature evidence from regional high-elevation tree-ring chronologies. A considerable amount of the variability in the temperature record may be linked to changes in the North Atlantic Oscillation.

**Electronic supplementary material:**

The online version of this article (10.1007/s00382-018-4555-y) contains supplementary material, which is available to authorized users.

## Introduction

One of the most pressing environmental concerns facing society today is climate change (IPCC 2014). Available evidence indicates that Earth is warming, but it is known that up to the past century many parts of the Northern Hemisphere were affected by a climatic deterioration lasting for hundreds of years that came to be called the Little Ice Age (LIA), a period which strongly affected the natural environment and human society (Lamb 1977; Grove 1988). During recent decades, the LIA has been a topic of major research efforts in paleogeography, paleoclimatology, dendrochronology and other disciplines. Presently, increasingly compelling evidence for a large-scale negative climate anomaly that occurred between the fifteenth/sixteenth and nineteenth centuries has become available from many parts of the globe and from various paleoclimatic archives, including ice cores, tree rings, borehole temperatures, lake sediments, glacier length records, and historical documents (Bradley and Jones 1993; Mann 2002; PAGES 2k Consortium 2013). The LIA cooling seems to have been driven mainly by volcanic forcing and possibly modulated by changes in solar activity as well as internal feedbacks in the climate system (Shindell et al. 2003; Miller et al. 2012; Schurer et al. 2014; Atwood et al. 2016). This pre-industrial period of dramatic climatic shifts deserves attention because of its importance for understanding of the underlying mechanisms that give rise to climate system variability and thus, for a deeper insight into modern and future climate change (Masson-Delmotte et al. 2013).

Progress in assessing the spatial extent, amplitude, and timing of the LIA resulted from numerous temperature reconstructions from continental to global scales (e.g. Moberg et al. 2005; Loehle 2007; Huang et al. 2008; Mann et al. 2008; Bertler et al. 2011; Christiansen and Ljungqvist 2012; PAGES 2k Consortium 2013; Neukom et al. 2014; Shi et al. 2015; Schneider et al. 2015; Luterbacher et al. 2016; Wilson et al. 2016; Xing et al. 2016), pointing to the coldest intervals of the LIA with mean summer/annual temperatures 0.5–1.0 °C lower than those of the twentieth century. The LIA cooling, however, was not continuous and uniform in space and time. It was heterogeneous in terms of its precise timing and regional dimension and this heterogeneity may be masked in climate reconstructions on a larger spatial scale (Matthews and Briffa 2005; PAGES 2k Consortium 2013). Therefore, a closer examination of LIA temperature records at various sites is needed to gain a deeper insight into the LIA climate variability at regional scales that is more relevant for ecosystems and human populations than globally averaged conditions.

Remote mountain lakes are closely coupled with atmospheric forcing factors and are largely considered to be affected by global scale processes, such as climate change, acting at a regional scale (Catalan et al. 2013). Providing a variety of information-rich signals through their physical, chemical, and biological responses to climate, these lakes offer significant opportunities for acquiring paleoclimatic data (Williamson et al. 2009). In high-alpine environments where the sensitivity to climatic change may be paramount (Pepin et al. 2015), factors other than temperature often are much less important in affecting species composition in lakes (Lotter and Psenner 2004). Of the various biotic proxies preserved in sediments of these lakes, the chitinous larval remains of chironomids (non-biting midges, Insecta: Diptera: Chironomidae) are one of the most important proxy indicators for reconstructing past climate change (Bradley 2015). Chironomids have relatively short life cycles, which are generally completed within 1 year (Eggermont and Heiri 2012), and can be expected to respond rapidly to changes in their environment. Species with narrow thermal tolerance (stenothermal) may be particularly vulnerable to changing climatic conditions, being potentially good indicators of climate change (Marziali and Rossaro 2013). Evidence from studies over the last decades in Eurasia and North America (Brooks 2006; Walker and Cwynar 2006) overwhelmingly points to summer temperature as one of the dominant drivers governing the distribution and abundance of lacustrine chironomid species. The development of transfer functions based on the modern distribution of chironomids among a large set of lakes (e.g. Barley et al. 2006; Heiri et al. 2011; Zhang et al. 2017) allows to infer summer temperatures from past changes in chironomid assemblages (e.g. Heiri et al. 2003; Ilyashuk et al. 2011; Samartin et al. 2017).

In the European Alps, the LIA is well documented in historic records with a rich set of evidence of large temperature anomalies, heavy winter snowfall, and a general advance of alpine glaciers (Fagan 2002). A number of studies have been concerned with reconstructing temperature variability in the Alps over centuries to millennia, including the LIA period. Despite the potential of chironomids to infer past temperature change effectively, the number of robust chironomid records available for this time period is still limited. Valuable temperature reconstructions producing smoothed estimates for a large spatial area have been derived from tree-ring networks, which are largely represented by high-elevation sites from the Western Alps (Büntgen et al. 2005, 2006; Frank and Esper 2005; Corona et al. 2011) and the southern fringes of the Eastern Alps (Corona et al. 2010; Coppola et al. 2013). Multi-archive temperature reconstructions have been developed from the combination of early instrumental temperature time series and documentary evidences (Casty et al. 2005), and tree-ring and lake sediment data (Trachsel et al. 2012). High-resolution millennium-long temperature reconstructions based on various proxies, including chironomids, are available from varved sediments of Lake Silvaplana, the south-eastern Swiss Alps (Larocque-Tobler et al. 2010; Trachsel et al. 2010; de Jong et al. 2013). Chironomid-based temperature records with a LIA signature have also been obtained from the northern French Alps (Millet et al. 2009) and the northern Swiss Alps (Larocque-Tobler et al. 2012). In the Austrian Alps, a temperature drop associated with the LIA has been documented in a speleothem oxygen isotope record (winter temperatures; Mangini et al. 2005), a diatom based record from lake sediment (spring/autumn temperatures; Schmidt et al. 2007) and tree-ring data (summer temperatures; Nicolussi et al. 2004; Esper et al. 2007; Büntgen et al. 2011; Klusek et al. 2015). All these reconstructions provide important insights into the temporal and spatial pattern of temperature anomalies during the LIA in the Alpine region. The combined information of published summer temperature reconstructions from the Alpine region shows a generally coherent picture of relatively cold conditions in the 17th and early nineteenth centuries. The available proxy archives, each with particular strengths and limitations in representing climatic variability (Huntley 2012; Christiansen and Ljungqvist 2017), are not evenly distributed, however, and substantial uncertainties with regard to the development of the LIA in individual regions of the Alps still exist. A strong topographic complexity, environmental features of the different mountain areas and the competing influences of a number of different climate regimes in the region (Beniston 2006) pose particular challenges in reconstructing the spatial pattern of climate processes through time, especially for the high-alpine region above 2200–2400 m elevation. Hence, obtaining new robust temperature proxy records is crucial to broaden our knowledge of the temporal and spatial extent of climate deterioration during the LIA across the Alpine region.

Here, we present the first 700-year-long high-resolution (4–10 years) record of mean July air temperature inferred by chironomids from a high mountain lake located in the Stubai Alps, one of the largest mountain chains of the Eastern Alps. A pattern of chironomid assemblage changes in this lake through the past 700 years has been described in detail earlier (Ilyashuk et al. 2015). The principal aim of this study was to provide chironomid-based summer temperature estimates, independent from tree ring chronologies, and to determine the main features of the multi-century climate development in this alpine region by giving emphasis to the time interval corresponding to the LIA. We compare our results with existing paleotemperature records from the Alpine region and other areas of the Northern Hemisphere in order to provide a large-scale context for understanding the pattern of temperature changes reconstructed in our study. We also compare the chironomid-inferred temperature variations with large-scale climate forcing processes to evaluate their role in driving temperature changes at the high-alpine site. Further, we assess inferred temperatures through comparison with instrumental temperature data and discuss potential sources of uncertainty to validate the reliability of the reconstruction.

## Materials and methods

### Study area

**Fig. 1:**
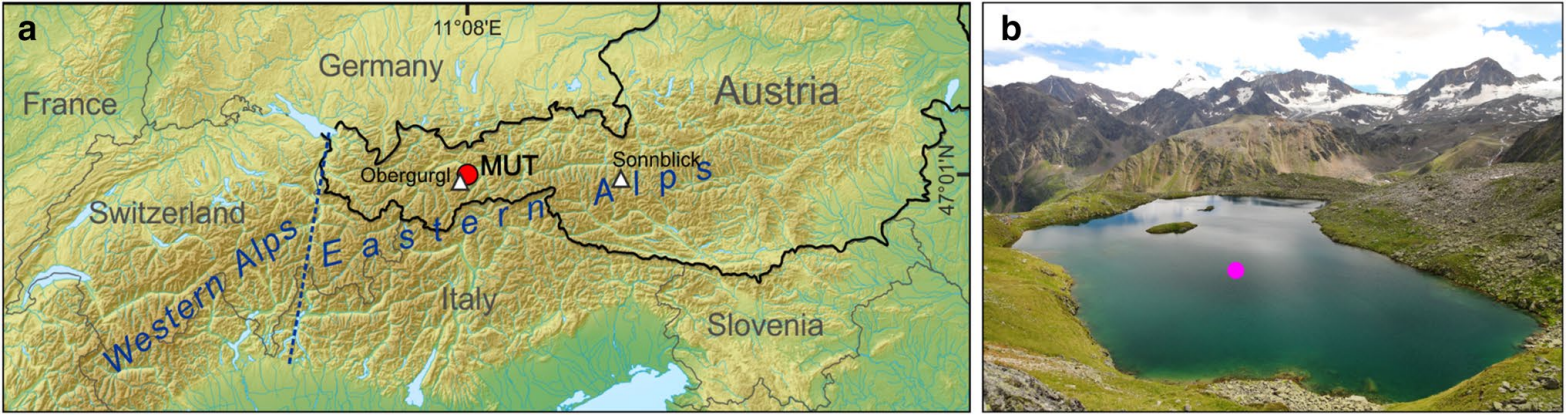
**a** Overview map showing the location of Lake Mutterbergersee (MUT) and the weather stations Obergurgl–Vent and Sonnblick in the European Alps. **b** Photograph of MUT and its catchment (looking towards the south) showing the coring site (closed circle)

Lake Mutterbergersee (MUT; 47°01′N; 11°08′E; 2483 ma.s.l.) is a lake located above the treeline on a south-east facing steep slope of the Stubai valley (the central valley of the Stubai Alps) that is a site of the Long-Term Ecological Research network in the Tyrol region, Austria (http://www.lter-austria.at/stubai/). This small lake covers an area of 3.8 ha and has a maximum depth of 8.0 m. The lake is oligotrophic with a concentration of total phosphorus of 3.7 µg/l (September 2010). The basin lacks well-developed inflows and outflows, and its glacier-free catchment is limited to ca. 20.0 ha, which means that lake-water exchange and lake-level fluctuations are determined by local groundwater conditions. Temperature stratification during the ice-free period is weak but stable with episodic stormy periods in summer when the upper layers can be mixed (Rott 1988). The lake has no permanent settlements in the immediate vicinity and is almost undisturbed by direct human activities (Fig. 1). The remote lake has been in the focus of paleolimnological investigations (Koinig et al. 1998; Ilyashuk et al. 2015) that have shown that the lake has continuous sedimentation of fine material in the deepest part and its biotic components reacted very sensitively to changes in the external environment, thus suggesting that its chironomid assemblages can be useful for deciphering past temperature changes based on the sedimentary archive from this lake.

The present climate at MUT is characterized by the mean annual air temperature close to 0 °C and a high average number of days per year with negative temperatures. These conditions cause prolonged ice cover on the lake, which can last from October to June. Over the period 1961–1990, the official climate normal period defined by the World Meteorological Organization (Baddour 2011), we estimated the mean monthly air temperatures by correcting the air temperatures measured at the nearby (18 km) Obergurgl–Vent meteorological station (1938 m.a.s.l.; http://www.zamg.ac.at/histalp; Auer et al. 2007) to the lake elevation and using surface air temperature lapse rates in the Tyrolean Alps (Rolland 2003). The computed air temperatures at the MUT site range from − 7.2 °C in January to 7.5 °C in July, with an annual mean of − 0.1 °C.

### Lake sediment sequence and chironomid record

A 32.6 cm long sediment core was retrieved at the deepest point of MUT using a gravity corer (UWITEC Ltd., Austria) in September 2010 and sectioned contiguously at 0.22-cm increments using an UWITEC core cutter with space laminae of 0.22 cm thick. For constructing age-depth relations of the samples, we refined the chronology developed by Ilyashuk et al. (2015), which is based on 16 ^210^Pb activity determinations and 3 ^14^C dates derived from terrestrial plant macrofossils, through producing a new Bayesian chronological model with updated parameters (see SI 1 for further details). The resulting age-depth model indicates that the core exhibits a mean sedimentation rate of 0.46 mm/year and spans the past 700 years (Fig. S1). The average temporal resolution of the 0.22-cm sediment layers is ca 4.8 years.

Subfossil chironomid analysis was performed on 100 sediment samples of 0.22 cm thickness, following standard procedures (see SI 2 for further details). Every sample was analyzed at the top 11.5 cm of the core (resulting in the temporal resolution of the core interval of 4–5 years), and every other one at the lower part (resulting in the temporal resolution of the interval of ca 10 years). Chironomid assemblage zones were determined through constrained optimal sum of squares partitioning and the number of zones was assessed statistically by the broken stick model using the Psimpoll 4.27 software (Bennett 2009). The main directions of variation in the down-core chironomid data were explored by calculating sample scores along principal components analysis (PCA) axes, following a square-root transformation of the taxon percent-abundance data to optimize the ‘signal’ to ‘noise’ ratio in the data. Only chironomid taxa present at ≥ 2% relative abundance in at least one of the samples analysed were included in the analysis. PCA was run with the CANOCO 5.03 software package (ter Braak and Šmilauer 2012). The statistical significance of the PCA axes was assessed by comparison with the broken-stick model (Legendre and Legendre 1998).

### Paleotemperature inferences

Quantitative mean July air temperature estimates were produced using a chironomid-temperature transfer function based on a modern calibration dataset consisting of chironomid assemblage data from 274 lakes in the Alpine and Norwegian regions (Heiri et al. 2011). This combined dataset encompasses a larger temperature range (3.5–18.4 °C) and greater taxonomic diversity (154 chironomid taxa) than most other existing regional European calibration datasets. Providing suitable analogues for many late Quaternary fossil sequences and a good concordance between reconstructions (Heiri et al. 2014a), this joint calibration set has been widely used in recent years to reconstruct past temperature change across Europe. The inference model is based on a two-component weighted averaging partial least squares regression and predicts mean July air temperature within the calibration data with a bootstrapped root mean square error of prediction (RMSEP) of 1.40 °C for predicting absolute temperatures and a coefficient of determination between inferred and observed July air temperature values of 0.87. The model is described in detail in Heiri et al. (2011). Temperature reconstructions were implemented with the software package C2 version 1.5.0 (Juggins 2007). Given that in the chironomid-temperature calibration data set the mean July air temperature is closely correlated with mean summer (June–August) temperature, and since it can be expected that chironomid assemblages actually respond to temperature over the entire growing season rather than to temperature within a particular month (Eggermont and Heiri 2012), the reconstruction can be considered to be representative for long-term changes in summer air temperature in general. To assess the relationship between the reconstructed variable and variations in chironomid assemblages, the inferred temperatures and the chironomid PCA axis 1 scores were compared by computing the Pearson product-moment correlation coefficient (*r)*. A clear correlation between the chironomid-inferred temperature values and the chironomid PCA axis 1 scores may suggest that the reconstructed variable has been exerting a significant influence on the MUT chironomids.

To detect statistically significant breakpoints (trend changing points) and periods with significantly different trends in the temperature record, we conducted segmented (piecewise) regression analysis using the SegReg software (Oosterbaan 2011). A possible breakpoint year, with a confidence interval, was estimated and separated periods of significant trend change before and after the breakpoint were determined based on maximizing the statistical coefficient of explanation and passing tests of significance applying a minimum confidence level of 95%. A nonparametric trend test including the Mann–Kendall’s tau (τ) statistics (Kendall 1975) in conjunction with the Theil-Sen slope estimator (Sen 1968) was used to detect statistically significant monotonic trends in the temperature record before and after a breakpont and to quantify the magnitude of identified linear trends. The significance levels of the test statistics were estimated using a block bootstrapping approach in order to account for the serial correlation present in the time-series data (Kundzewicz and Robson 2000). The Kendall-Theil method for finding the slope of a trend line is based on medians, and, therefore, robust against outliers. The software programs TREND 1.2 (Chiew and Sirivardena 2005) and Kendall-Theil Robust Line 1.0 (Granato 2006) were used in the calculations.

### Inferred temperatures: their reliability and comparison to other proxy records

We applied a series of numerical analysis procedures to assess whether the reconstructed temperature values were well supported by the calibration data and the applied transfer function. The representativeness of the down-core chironomid assemblages in the modern calibration data set was explored by applying the modern analogue technique (MAT) with Chi square distance as the dissimilarity measure. Samples with values below the 2% cut-off level of all distances in the modern calibration data were defined as having ‘close’ modern analogues (Birks et al. 1990; Heiri et al. 2003). Sample specific prediction errors (SSPEs) were calculated for the individual down-core samples using bootstrapping (9999 bootstrap cycles; Birks et al. 1990). Analogue statistics and sample-specific errors of prediction were calculated using the program C2 version 1.5.0 (Juggins 2007). The goodness of fit of down-core chironomid samples to temperature was tested by a canonical correspondence analysis (CCA) of the modern training set samples constrained solely by July temperature and the down-core samples positioned passively on the CCA ordination implemented in CANOCO version 5.03 (ter Braak and Šmilauer 2012). Samples with a residual distance to the first CCA axis above the 90th percentile of values in the training set samples were considered to have a ‘poor’ fit to temperature (Birks et al. 1990).

To test the relationship between chironomid and mean July air temperature changes over the instrumental period, we examined structural changes in the chironomid assemblages as summarized by PCA axis 1 sample scores and the chironomid-inferred temperature variations at MUT against the instrumental temperature records from: (1) the closest weather station Obergurgl–Vent, (2) Sonnblick, the highest weather station in Austria, and (3) the homogenized CRSM-series (Coarse Resolution Subregion Mean) of temperature for the high-elevation subregion ALPIN (Auer et al. 2007; Brunetti et al. 2009). All data were obtained from the HISTALP project database (http://www.zamg.ac.at/histalp/). The relationships were tested with Pearson’s *r*. The significance of the relationships were verified using bootstrap estimates of 95% confidence intervals (95% CI_boot_) for coefficient values based on the bias corrected and accelerated bootstrap procedure with 2000 iterations. Correlations were deemed statistically significant at the 95% level if the confidence intervals did not include zero. This nonparametric technique randomly re-samples blocks of data pairs with replacement to account for the presence of autocorrelation in the time series and offers robustness against data distribution and time spacing (Mudelsee 2003). The SPSS Statistics software version 23.0 (IBM Corp., Armonk, USA) was used in the calculations. Since the chironomid-based reconstruction has a temporal resolution of about 4–5 years at this time interval, a Gaussian low-pass filter with 5-year cutoff periods was applied to all temperature records before comparisons. For a better comparison, we considered the time windows that overlap with each instrumental record and an overlapping interval common to all time series (AD 1887–2008).

The general pattern of chironomid-inferred temperature changes extending back to AD 1300 was compared with regional- and large-scale surface temperature reconstructions. To highlight the longer-term (multi-decadal) variability and facilitate comparison, the Pearson’s correlation coefficients were calculated after 30-year Gaussian low-pass filtering and linear detrending the data sets.

## Results and discussion

### Main patterns of temperature variability in the record

**Fig. 2:**
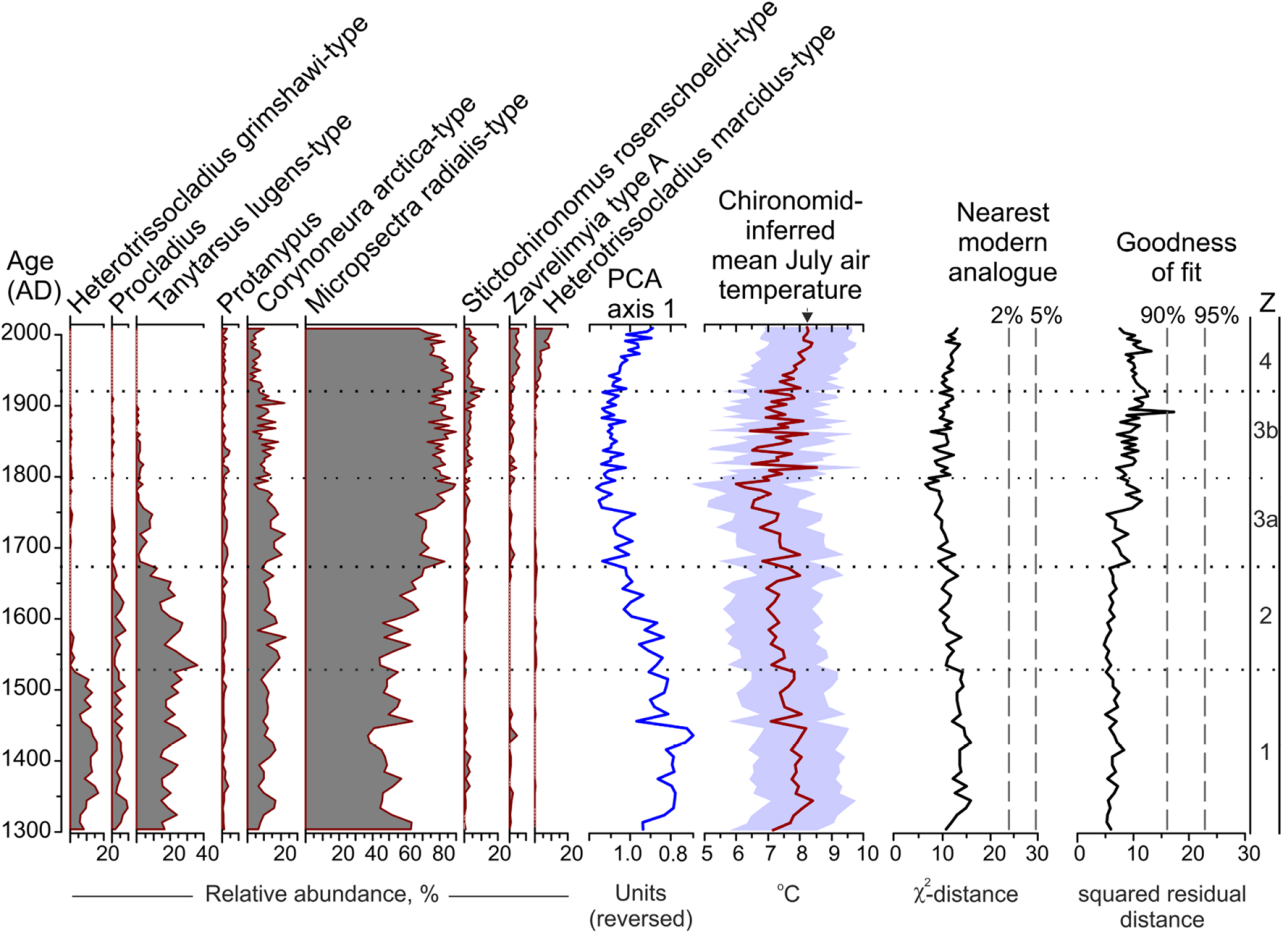
Chironomid diagram for MUT showing the relative abundance (%) of the most common taxa, the sample scores of PCA axis 1 and chironomid-inferred mean July air temperature plotted together with sample-specific prediction errors (SSPE, colored envelope). Boundaries between statistically significant assemblage zones (Z) as well as between subzones 3a and 3b are marked by dotted lines. Reconstruction diagnostic statistics such as nearest modern analogues for the down-core samples in the training set data, and goodness-of-fit of the down-core samples with temperature are indicated. Vertical dashed lines are used to identify samples with no ‘close’ (2%) modern analogues and samples with ‘poor’ (0.90) fit with temperature (see text for details). The black triangle indicates the elevation corrected instrumental average July air temperature (8.2 °C) from the weather stations Obergurgl–Vent and Sonnblick for the period 2006–2010 covered by the uppermost sample

**Fig. 3:**
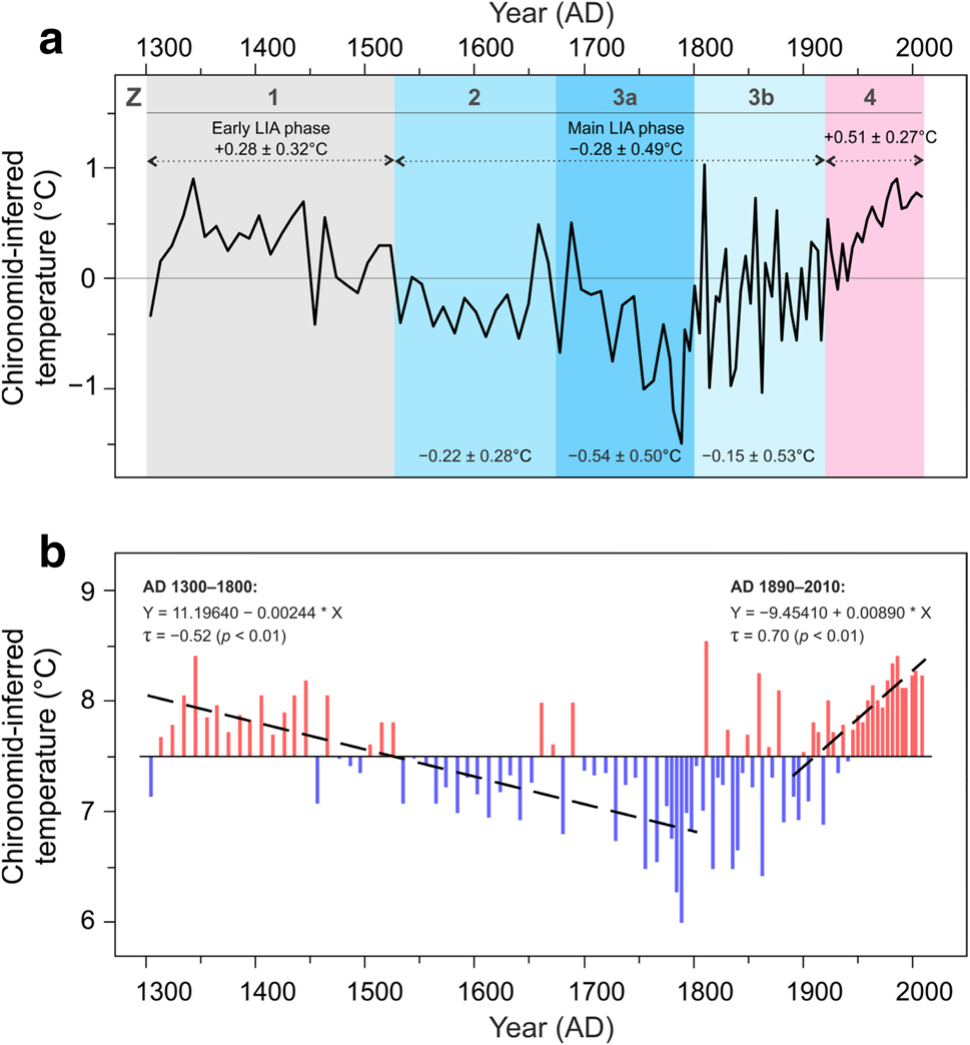
Chironomid-inferred mean July air temperature variability at MUT. **a** Temperature anomalies compared to the mean value over the entire record (7.5 °C). The numbers are the average temperature anomaly for time intervals corresponding to the zones (Z) and subzones established in the chironomid stratigraphy. **b** The linear decreasing (AD 1300–1800) and increasing (AD 1890–2010) trends of temperature values calculated by the Kendall-Theil robust line (dashed lines). The trend line equations with the median slope (°C/year) and intercept, the Mann–Kendall’s tau (τ) coefficient and the two-tailed *p* values are also shown

The chironomid-based reconstruction of mean July air temperatures from MUT documents four distinct phases of temperature variability in the Austrian Alps over the past 700 years, as defined by the statistically significant zonation of the chironomid stratigraphy (Ilyashuk et al. 2015; Figs. 2, 3a). In addition, taking into account that PCA axis 1 summarizing the major variance in the chironomid data has the highest sample scores near AD 1800, the boundary between subzones 3a and 3b was delineated visually based on clearly contrasting patterns of PCA axis 1 change before and after ca AD 1800 (Figs. 2, 3a). A summary diagram of the chironomid stratigraphy showing the relative abundance (%) of all taxa present in the lake during the past 700 years and the established zonation is given in Fig. S2. At the beginning (Zone 1, AD 1300–1530) and at the end (Zone 4, AD 1920–2010) of the record, the reconstruction indicates slightly elevated temperatures (+ 0.28 ± 0.32 °C and + 0.51 ± 0.27 °C, respectively) relative to the 700-year mean (7.5 °C), while inferred temperatures are generally cooler (− 0.28 ± 0.49 °C; Zone 2 and Zone 3, AD 1530–1920) between those two warmer periods (Fig. 3a).

This chironomid-based evidence for a prolonged period of predominantly cooler summer conditions during AD 1530–1920 is temporally in close agreement with the climatically defined LIA in Europe, namely the main LIA phase between about AD 1550 and 1900 (Jones et al. 2001; Matthews and Briffa 2005; PAGES 2k Consortium 2013). The period between about AD 1250 and 1550 has been regarded as the early LIA phase, or as transition from the Medieval Warm Period (MWP) to the main LIA phase (Summerhayses 2015). The main LIA phase at MUT (AD 1530–1920) appears to have consisted of two cold time intervals divided by slightly warmer episodes in the second half of the seventeenth century. Although these warmer intervals are very brief, a similar structure of the LIA, with a return to somewhat warmer conditions, is visible in several reconstruction from the Alps (Trachsel et al. 2010; Larocque-Tobler et al. 2010, 2012), Scandinavia (Zawiska et al. 2017), Greenland (Kobashi et al. 2011), other regions of Europe (Luterbacher et al. 2016), and the entire Northern Hemisphere (Mann et al. 2009). Yet, the LIA is characterized by a considerable spatial and temporal expression of climate variability and the debate about the exact onset and termination of the LIA remains heavily dependent on the proxies used, their geographical locations, reconstruction methods and temporal resolution of proxy records (PAGES 2k Consortium 2013).

The segmented regression analysis applied to the MUT chironomid-based temperature reconstruction revealed significant trend changes (breakpoints) near AD 1800 and in the early AD 1890s (see SI 3 and Fig. S3 for further details). The Mann–Kendall test confirmed the presence of two statistically significant (p < 0.01) trends in the record. The reconstructed temperatures show a well-marked decreasing trend (τ = −0.52) from AD 1300 until about AD 1800 (− 1.2 °C in 500 years) (Fig. 3b). Figure 3 demonstrates that the transition into the coldest phase of the LIA after AD 1530 is marked by a drop in the frequency of decadal scale intervals in which the chironomid *Heterotrissocladius grimshawi*-type, typical for slightly warmer temperatures, was more abundant. The subsequent period AD 1800–1890, with no uniform temperature trend, is characterized by substantial sub-decadal variability with a range of up to 2 °C between warm peaks and cold troughs. The increased variability during this period may be inferred as a result of an increased number of rare taxa due to the decreasing abundance of some chironomids having been common before the coldest phase (*Heterotrissocladius grimshawi*-type, *Procladius* and *Tanytarsus lugens*-type), and may be taken as a sign of ecosystem reorganization induced by a shifting climate pattern. A continuous upward trend (τ = 0.70) was identified since AD 1890 until the end of the record at AD 2010 (1.1 °C in 120 years). After AD 1920, the inferred temperatures were generally higher than the long-term mean, resulting from the increase in the taxa adapted to warmer conditions (*Heterotrissocladius marcidus*-type, *Stictochironomus rosenschoeldi*-type and *Zavrelimyia* type A) in the uppermost layers.

**Fig. 4:**
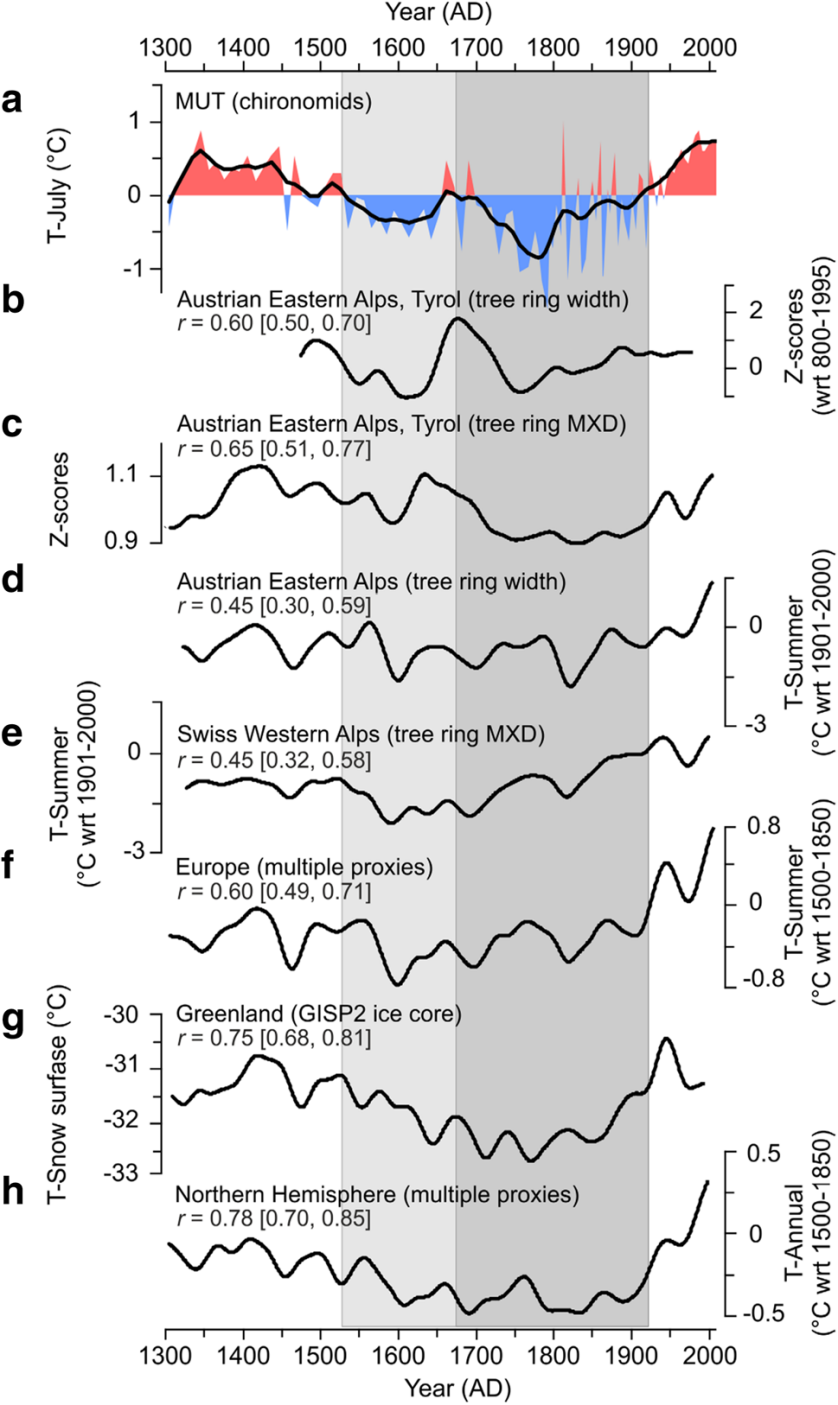
Temperature reconstructed in this study compared with other 30-years low-pass filtered paleotemperature records. **a** Chironomid-inferred mean July air temperature at MUT plotted along with the smoothed curve (thick line) centered to zero mean over the entire record, **b** tree-ring width (Nicolussi and Schiessling 2001; Osborn and Briffa 2006) and **c** maximum latewood density (MXD; Esper et al. 2007; Schneider et al. 2015) chronologies from Tyrol, Austria; mean summer air temperature inferences based on **d** tree-ring width chronologies from the Austrian Eastern Alps (Büntgen et al. 2011) and **e** tree-ring MXD chronologies from the Swiss Western Alps (Büntgen et al. 2006), **f** European mean summer temperature variability derived from tree-ring width/MXD and documentary records (Luterbacher et al. 2016), **g** average snow surface temperatures reconstructed from the GISP2 ice core near the Summit of the Greenland ice sheet (Kobashi et al. 2011), and **h** Northern Hemisphere mean annual air temperature reconstructed using multiple proxies (errors-in-variables approach; Shi et al. 2013). Pearson correlation coefficients (*r*) between chironomid-inferred mean July air temperature at MUT and other proxy-based temperature records (all after applying a 30-year Gaussian low-pass filter and linear detrending) are shown; the 95%-confidence intervals obtained by block bootstrap resampling are given in square brackets. The grey areas correspond to the colder periods identified in the MUT temperature reconstruction. *wrt* with regard to

The inferred multi-centennial pattern of temperature changes at MUT is generally consistent with previously obtained summer temperature records from the European Alps, which are based on regional-scale tree-ring network compilations (Büntgen et al. 2005, 2006, 2011; Frank and Esper 2005; Corona et al. 2010) or the combination of tree-ring chronologies with lake sediment data (Trachsel et al. 2012). Some differences do exist at multi-decadal to centennial scales. Stacked reconstructions based on a number of individual tree-ring records, largely from the Western Alps, show the coldest decades of the LIA in the 17th and the early nineteenth centuries, which matches two main phases of glacier advances in this region during the seventeenth and nineteenth centuries (Holzhauser et al. 2005). Such a temperature development is supported by chironomid-based temperature reconstructions from varved lake sediments in the Swiss Western and Eastern Alps (Larocque-Tobler et al. 2010, 2012). Our temperature record from the high-elevation site in the Austrian Eastern Alps indicates a steady decline in temperatures through the eighteenth century, characterized by a sequence of extremely cold decades, to a minimum around AD 1780–1790 with the lowest temperatures in the record (Fig. 3). The temperature depression is coeval with the reported maximum extent of most East Alpine glaciers at ca AD 1780 (Rott 1993; Nicolussi 2013). Cold summers during much of the eighteenth century are also clearly observed in high-elevation stone pine tree-ring chronologies from the Austrian part of the Eastern Alps (Fig. 4) (Nicolussi and Schiessling 2001; Nicolussi et al. 2004; Esper et al. 2007) used in the reconstructions dealing with spatial summer temperature variability over the past five centuries in the Greater Alpine Region (Wilson et al. 2005) and millennium-long, hemispheric scale temperature variations (Esper et al. 2002; D’Arrigo et al. 2006; Osborn and Briffa 2006; Schneider et al. 2015).

Some discrepancies identified between the temperature records from the Western and Eastern Alps can be ascribed to the spatial variability of climate in the Alps resulting naturally from the diversity of topographical conditions and the physical processes governing atmospheric variability. Continentality is one of the major factors governing the Alpine climate (Beniston 2006). While the western part of the mountain chain is more strongly influenced by the temperate Atlantic climate, the Central Eastern Alps are characterized by a more continental climate and consequently more extreme weather conditions (Efthymiadis et al. 2006). Distinct climatic gradients, in all three dimensions of space, are a prominent feature of the Alpine climate (Gobiet et al. 2014) and, therefore, even small differences in longitude, latitude and topographic relief within the Alpine region are associated with substantial differences in climate trends. In addition, different sensitivities of taxa and groups of organisms (e.g. terrestrial vs. aquatic organisms; Wilson et al. 2015; Shala et al. 2017) used as natural proxies for temperature inferences may also be responsible for differences between regional temperature records.

The inferred pattern of temperature changes in the Central Eastern Alps is generally consistent with many other temperature reconstructions on a broader geographic scale than the regional one. For example, the MUT temperature development shows a remarkable likeness to a chironomid record (PCA axis 1 sample scores) from Iceland, which is interpreted as an expression of summer temperature variability and provides evidence of a temperature drop at ca AD 1535 followed by a steady decline through the seventeenth and eighteenth centuries to a minimum around AD 1780 (Holmes et al. 2016). The MUT temperature reconstruction agrees well with millennium-long paleoclimate inferences based on ice core records from Greenland (Kobashi et al. 2011), Svalbard (Divine et al. 2011), and the Russian Arctic (Opel et al. 2013), all of which identify the most pronounced cooling events in the eighteenth century. Similar to the MUT record, reconstructions derived from global borehole temperature measurements (Huang et al. 2008) recorded the lowest temperatures during the eighteenth century, with a LIA temperature minimum about 1.2 °C below the MWP maximum, and about 1.7 °C below present-day temperatures. Figure 4 demonstrates that on a multi-decadal time-scale (30 years) the mean July temperature record from MUT exhibits a fair degree of coherence with temperature reconstructions obtained on the regional and broader geographic scales, European and Northern hemispheric. Still in some records earlier phases of the LIA are cooler than the eighteenth century which represents the coolest period in the MUT record. As reviewed by Christiansen and Ljungqvist (2017), apart from the agreement in the general form of the low-frequency variability quantified by pairwise correlations between 30 years smoothed reconstructions, the centennial-scale amplitude of the LIA often differs in proxy records and large-scale temperature reconstructions, being attributed to the reconstruction methodology and proxy selection strategy.

### Responses to large-scale climate forcing processes

**Fig. 5:**
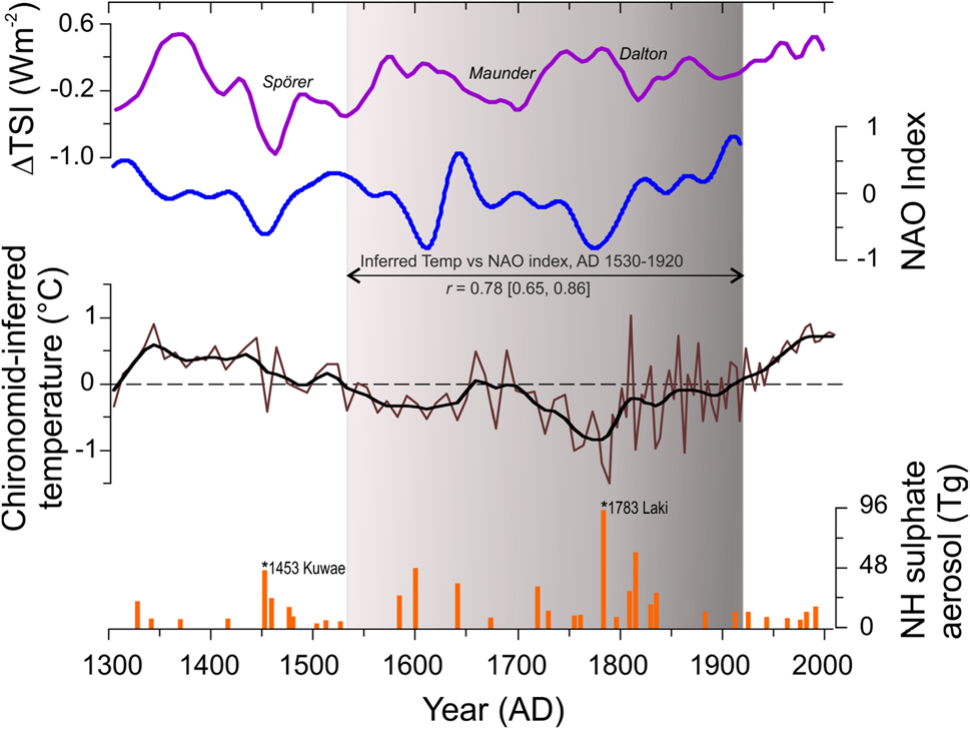
Temperature record from MUT compared with large-scale climate forcing processes such as variations in total solar irradiance (∆TSI, Steinhilber et al. 2009), the North Atlantic Oscillation (NAO index, 30-years low-pass filtered, Ortega et al. 2015), and the Northern Hemisphere (NH) stratospheric aerosol loading from explosive volcanic eruptions (Gao et al. 2008). Chironomid-inferred mean July air temperature for MUT is presented as original (thin line) and 30-years low-pass filtered (thick line) data and expressed as departures from the long-term average for the entire record. Pearson *r* calculated between chironomid-inferred mean July air temperature at MUT (Inferred Temp) and NAO index for the main LIA phase (AD 1530‒1920) after applying a 30-year Gaussian low-pass filter and linear detrending is shown; the 95%-confidence intervals obtained by block bootstrap resampling is given in square brackets

Quantifying climate forcings and feedbacks over the last millennium in climate model simulations indicates that the LIA cooling in the Northern Hemisphere was largely driven by volcanic forcing, while a contribution due to changes in solar insolation was substantially lower (Schurer et al. 2014; Atwood et al. 2016). For example, periods of cooling in the Northern Hemisphere after major volcanic eruptions of the last two centuries usually last for 4 years (Self et al. 1981), whereas multiple volcanic eruptions closely spaced in time (phases of increased volcanism) have the potential to affect climate over decadal to multi-decadal time frames (Crowley 2000; Zielinski 2000; Miller et al. 2012; Robock 2015). In the MUT record, the LIA temperature minimum of 1.5 °C below the long-term mean (an offset larger than the prediction error of the model) is recorded in two adjacent samples dated to the mid-1780s. This minimum may coincide within dating uncertainties with the largest volcanic signal of the last 700 years (Fig. 5) detected in Greenland ice cores, which is assigned to the long-lasting AD 1783–1784 Laki eruption in Iceland (Gao et al. 2008). Uncertainties associated with the dating of sediment records do not allow us to state this with certainty, but the possible connection between the temperature excursion and the volcanic event may have been amplified by the elevation of the lake, since high-elevation ecosystems are closely influenced by atmospheric forcing factors, and in these situations both aquatic and terrestrial life stages of chironomid midges are exceptionally susceptible to climatic change. This 8-month eruption was different to all major tropical eruptions in terms of the dynamical response and had considerable effects on climatic conditions around the Northern Hemisphere and the entire globe for years afterwards due to the direct radiative effects of the Laki aerosols and shifts in atmospheric circulation (D’Arrigo et al. 2011). Temperature records from Europe and the north-eastern United States show that the winter 1783–1784 was one of the most severe ones in the last 250 years and the summer temperatures during the 3 years following the eruption were cooler, especially in Europe, with values about 1.4 °C lower than the average for a 31-year period (AD 1768–1798) centered on AD 1783 (Thordarson and Self 2003). This is supported by gridded instrumental data from high-elevation sites in the Alps (Böhm et al. 2001) indicating that the July of AD 1786 was the coldest within the 30-year period AD 1780–1809.

The regional climate responses to perturbations caused by large volcanic eruptions are determined to a large extent by feedbacks via atmospheric/ocean dynamics and cold summers can be maintained by these feedbacks long after volcanic aerosols are removed (Miller et al. 2012). Large eruptions can produce decadal-scale shifts in the North Atlantic Ocean circulation (NAO) (Slawinska and Robock 2018). While the Alps are situated in a band of rather weak and varying forcing of the NAO, the dominant mode of winter climate variability in Europe (Hurrell et al. 2003), high-elevation sites in the Alps are sensitive to changes in the NAO (Beniston et al. 1997; Casty et al. 2005). A strong, coherent impact of the NAO on European lakes has been reported by a number studies (e.g. Straile et al. 2002; Livingstone and Dokulil 2001). The NAO affects temperature regime and ice phenology of lakes, and as a result these responses influence the length of the growing season and the population dynamics of freshwater organisms in summer (Straile et al. 2003). The comparison of chironomid-inferred temperatures with an independent reconstruction of past NAO variability (Ortega et al. 2015) shows a close agreement (Fig. 5), suggesting that considerable parts of the variability in the MUT summer temperature record during the LIA may be linked to changes in the NAO. The temperature changes between AD 1700 and 1800, the coolest period in the record, match particularly clearly to the negative NAO index phase associated with decreased westerlies over Western Europe. Also, a distinct cold pulse in the reconstructed temperatures in the 1450s, which matches the very cold decade detected in Alpine tree-ring records (Büntgen et al. 2006, 2011) and many summer temperature series across Europe (Camenisch et al. 2016; Zawiska et al. 2017), co-occurs with the prevailing negative NAO index as well as the minimum total solar irradiance that defines the Spörer Minimum (Fig. 5). In addition, two volcanic signals closely spaced in time in the decade of the AD 1450s (Fig. 5) suggest that explosive eruptions could have influenced the climate in that time, thus enhancing and possibly extending the cool climate existing at the time of the eruption (c.f. Zielinski 2000). There is evidence from historical and proxy data that the eruption of the submarine volcano Kuwae in AD 1453 in the tropical Pacific, one of the largest eruptions detected in ice cores from both polar regions (Gao et al. 2006; Cole-Dai et al. 2013), highly perturbed the atmospheric circulation in the mid-fifteenth century (Pang 1993; Briffa et al. 1998), confirming that volcanism can play a role in decadal-scale global or hemispheric cooling during the LIA (Zielinski 2000; Sigl et al. 2015).

### Reliability of the reconstruction

#### Reconstruction diagnostic statistics and comparisons with instrumental data

The temperatures reconstructed from MUT are well within the mean July air temperature gradient spanned by the calibration data, and all down-core taxa are well represented in the modern calibration data set. Also, all down-core samples have a ‘close’ modern analogue and a ‘good’ fit to temperature, except one sample (ca AD 1890) (Fig. 2). This suggests that the calibration data set covers past assemblage states in the MUT chironomid record well. The inferred mean July temperature of 8.2 °C for the topmost sample (representing the period AD 2006–2010) corresponds closely with the elevation corrected instrumental values from the weather stations Obergurgl-Vent (8.4 °C) and Sonnblick (8.0 °C) for this period. The offset of 0.2 °C clearly remains within the error limits of the model, supporting that the present chironomid assemblage composition represents the elevation and modern temperature conditions of the site well. A close correlation [*r* = − 0. 52 (− 0.63, − 0.39)] of the inferred temperatures with the scores of PCA axis 1, which is the only statistically significant axis explaining 85.4% of cumulative variance in the chironomid data, strongly suggests that a substantial proportion of the variability of the MUT chironomid assemblages is controlled by air temperature changes through direct as well as indirect ways. Indeed, the effects of regional temperature change on local chironomid assemblages are often indirect, and a variety of interacting environmental factors co-varying with temperature influence chironomid assemblages in lakes, determining the overall impact of climate change (Velle et al. 2010; Eggermont and Heiri 2012). As previously shown by Ilyashuk et al. (2015), cold-season temperature regime and lake ice phenology, as well as temperature-related changes in sedimentary properties are other important factors controlling the composition of the chironomid assemblages in MUT besides the direct effects of warm-season temperatures.

**Fig. 6:**
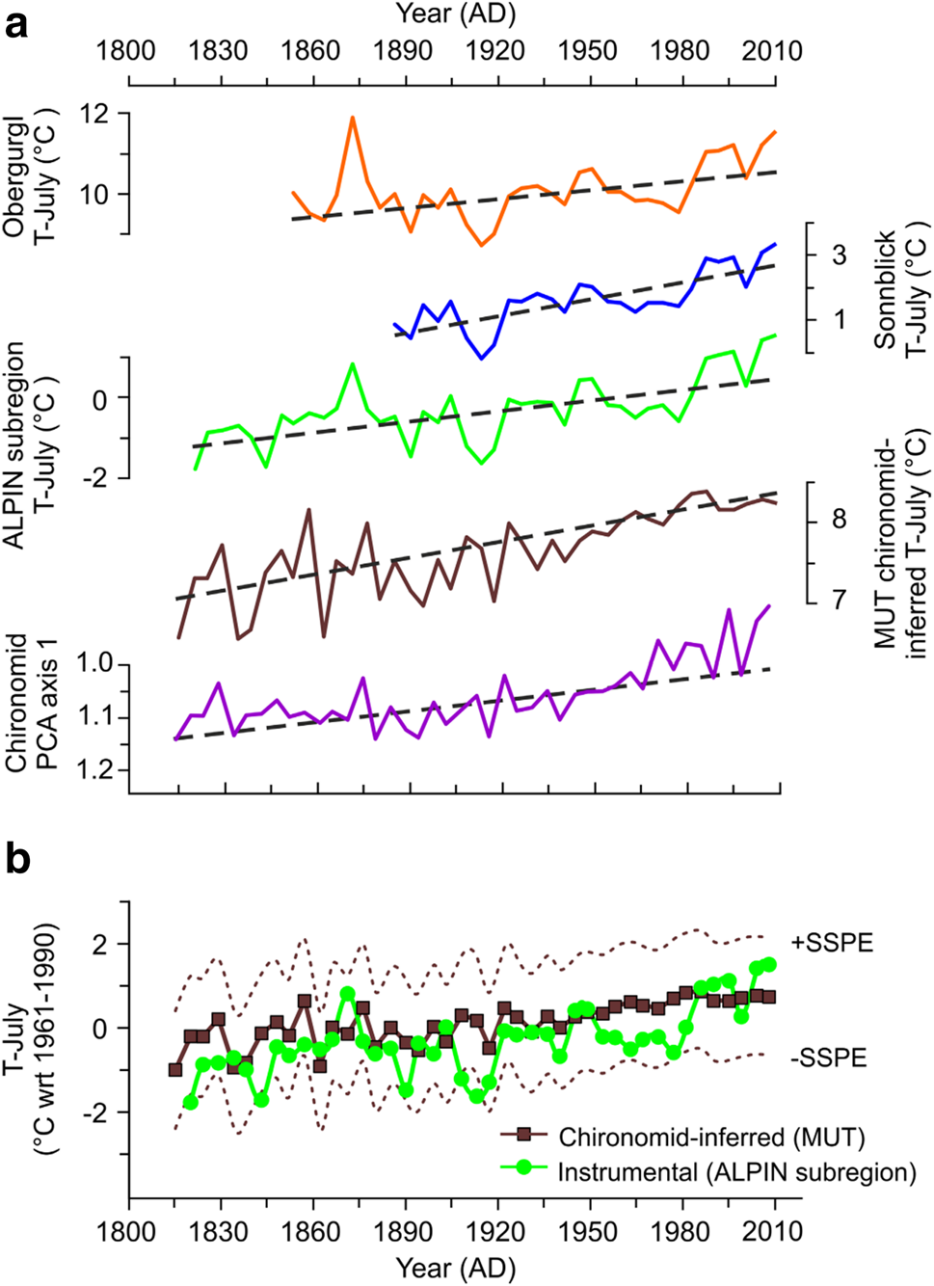
**a** Temperature variations reconstructed at MUT and chironomid PCA axis 1 sample scores compared with 5-years low-pass filtered instrumental temperature records (T-July). All records are plotted at the time resolution of the chironomid samples and together with monotonic trends (dashed lines) calculated using the Kendall–Theil robust line. In order to be comparable, the PCA axis 1 sample scores are plotted along the inverted *y* axis. **b** Chironomid-inferred July air temperatures from the MUT record (squares) plotted together with sample-specific prediction errors (SSPEs, dotted lines) compared with the multi-station reconstruction from the ALPIN subregion (circles). The values are anomalies from the 1961–1990 average. All instrumental temperature data are from the HISTALP project database (http://www.zamg.ac.at/histalp/)

An especially effective way to assess the performance of any reconstruction covering the instrumental period is a comparison to available long time series of meteorological data. Direct comparison of the chironomid PCA axis 1 sample scores and the chironomid-inferred temperatures with the instrumental temperature time series reveals substantial relationships for the sub-decadal (5 years) scale variations. The trends in chironomid PCA axis 1 sample scores and chironomid-inferred temperatures at MUT are consistent with upward trends in instrumental temperature series (Fig. 6a), which is also confirmed by the rather high correlation coefficients (Table 1). It is interesting to note that the correlations with the temperature time series from the Sonnblick weather station, the highest weather station in Austria, are higher than those with the temperature record from the nearest but lower elevated station Obergurgl-Vent (Table 1). This suggests that chironomids from our high-elevation site better reflect the temperature changes measured in remote mountain locations where a more pristine Alpine background climate persists and the signal coherency is not affected by local landscape characteristics and other factors related to elevation differences (Böhm et al. 2001).

**Table 1:** Pearson correlations (*r*) of the chironomid PCA axis 1 samples scores and chironomid-inferred mean July air temperatures at MUT (Inferred Temp) with the instrumental temperature records after applying a 5-years Gaussian low-pass filter

Instrumental data	Time interval overlapping with each instrumental record		Time interval common to all records AD 1887–2008 (*n* = 28)	
	PCA axis 1	Inferred temp	PCA axis 1	Inferred temp
	*r* (95% CI_boot_)	*r* (95% CI_boot_)	*r* (95% CI_boot_)	*r* (95% CI_boot_)
Obergurfgl-Vent	− 0.55 (− 0.81, − 0.21)	0.42 (0.06, 0.69)	− 0.67 (− 0.87, − 0.33)	0.56 (0.28, 0.76)
1938 m a.s.l				
1852–2008 (*n* = 35)				
Sonnblick	− 0.73 (− 0.86, − 0.48)	0.62 (0.39, 0.81)	− 0.73 (− 0.86, − 0.48)	0.62 (0.39, 0.81)
3105 m a.s.l				
1887–2008 (*n* = 28)				
HISTALP CRSM	− 0.66 (− 0.82, − 0,36)	0.54 (0.33, 0.70)	− 0.72 (− 0.87, − 0.46)	0.60 (0.37, 0.78)
ALPIN subregion				
1820–2008 (*n* = 42)				

The time windows that overlap with each instrumental record and an overlapping interval common to all time series (AD 1887–2008) are considered. Bootstrap 95% confidence intervals (95% CI_boot_) are given in square brackets

#### Potential sources of uncertainty in the reconstruction

Transfer function estimations of environmental variables, based on the relationship between taxa and the environment in a modern training set, have inherent sources of uncertainty arising when modeling a causal relationship (Birks et al. 2010). Variables other than summer temperature can affect chironomid assemblage composition, for example differences in in-lake productivity, nutrient and/or oxygen availability (Velle at al. 2010). However, under low intensities of human impact, as can be expected for high-alpine lakes such as MUT, these variables will themselves be strongly influenced and determined by climatic conditions (Eggermont and Heiri 2012). The MUT temperature reconstruction is presented with sample-specific prediction errors (1.37–1.44 °C) reflecting the uncertainty associated with calculating quantitative temperature estimates based on chironomid assemblage data. The relatively large RMSEP of the transfer function is one of the uncertainties which may limit our interpretation of the chironomid-inferred LIA coolings with amplitudes lower than the error of prediction. However, the comparison of the chironomid-inferred temperatures with homogenized mean July air temperatures for the high-elevation subregion ALPIN (the area-weighted mean altitude is 2402 m.a.s.l) over the time interval AD 1820 − 2008 reveals that most instrumental temperatures fall within the uncertainty expected for the applied transfer function (mean ± SSPE), and 90% of the inferences for this period had deviations from the instrumental data below the RMSEP (Fig. 6b). This agrees with the situation described for some other chironomid-based late Holocene records from the Alps in which even relatively small changes in the range of 0.5 °C are apparently faithfully reconstructed by chironomids (Larocque et al. 2009; Millet et al. 2009).

Late Holocene reconstructions from non-annually resolved sediment sequences are affected by chronological uncertainties inherent to radiocarbon dating and the construction of age-depth relationships (Telford et al. 2004). Analytical uncertainty in the generation of ^14^C dates, as well as wiggles and plateau in the ^14^C calibration curve, can cause large uncertainties on calendar ages if ^14^C dates are calibrated individually. Applying Bayesian age-depth modeling, as done to date the MUT record, can reduce this chronological uncertainty considerably, since all ^14^C dates are calibrated simultaneously, taking their stratigraphic order into account (Blaauw and Heegaard 2012). Dating uncertainties (2σ) in the resulting age-depth relationship were estimated to range from 2 to 8 years for the upper layers constrained by ^210^Pb dating (AD 2010–1874) to 14–74 years for layers older than AD 1874. Given that the MUT sedimentary record is continuous, without evidence for hiatuses or age reversals, we consider the age-depth model to give a reliable estimate for the age of the sediments.

Overall, the evaluation and validation results suggest that the chironomid-based temperature estimates at MUT are reliable back through time on multi-annual and longer timescales. The relatively high temporal resolution of the stratigraphic samples (ca 4.8 years) allows us to fix the timing of main LIA events and interpret proxy record inter-comparisons with some confidence. Uncertainties associated with proxy-based reconstructions can be reduced by cross-validation of different climate records and confirmation of inferred climatic patterns by several independent lines of evidence (Heiri et al. 2014b). Although the presented temperature reconstruction, derived from a remote mountain lake, is generally consistent with regional and large-scale records, other validations against additional chironomid records from the Eastern Alps are required to separate large-scale climate signals from record-specific variations and to confirm the robustness of this first late Holocene reconstruction. Also, further efforts to develop multiple, well-dated chironomid records for other regions of the Alps are essential to improve the ability to detect past changes in temperature gradients across the Alpine region (Heiri et al. 2015).

## Conclusions

The ultimate aim of this study was to broaden our knowledge of the spatio-temporal variability of summer temperature in the Alpine region, particularly during the LIA, by providing a new high-resolution 700-year long chironomid-based mean July air temperature record from the Austrian Alps. The record shows a notable decreasing trend of − 1.2 °C from ca AD 1300 until ca AD 1800 and provides evidence for a prolonged period of predominantly cooler conditions during AD 1530–1920, which is temporally largely equivalent to the climatically defined LIA in Europe. The main LIA phase at MUT appears to have consisted of two cold time intervals divided by slightly warmer episodes in the second half of the 1600s. An important feature of the record is a more steady and steep decline in temperatures through the eighteenth century. The LIA temperature minimum about 1.5 °C below the long-term mean recorded in the mid-1780 s coincides, within dating uncertainties, with the largest volcanic signal at AD 1783 (Laki eruption, Iceland) found in Greenland ice cores over the last 700 years and may be a manifestation of cooling that followed the eruption. A continuous warming trend (1.1 °C per 120 years) is evident since AD 1890 until the end of the record at AD 2010. After AD 1920 the inferred temperatures were generally higher than the long-term mean.

The multi-centennial pattern of chironomid-inferred temperature changes is generally consistent with previously obtained Alpine summer temperature reconstructions. The differences in temperature trends on multi-decadal to centennial scales can be attributed to the spatio-temporal variability of the Alpine climate resulting from the diversity of topographical conditions and the physical processes governing atmospheric variability. Comparison with large-scale reconstructions revealed that the temperature inferred from this high-elevation lake in the Alps generally follows broader-scale temperature variability in other European and Northern Hemisphere paleorecords. A considerable amount of variability in the MUT summer temperature record during the LIA may potentially be linked to changes in the NAO. Direct comparison of the reconstructed temperatures with the instrumental mean July temperature series reveals substantial relationships for the sub-decadal (5 years) scale variations.

## Electronic supplementary material

Below is the link to the electronic supplementary material.

Supplementary material 1 (PDF 1295 KB)
