# Supplementary material for: The Little Ice Age signature in a 700-year high-resolution chironomid record of summer temperatures in the Central Eastern Alps
Source: Clim Dyn. 2018 Dec 1;52(11):6953–67. doi: 10.1007/s00382-018-4555-y (PMC6534512; doi:10.1007/s00382-018-4555-y)
Supplement: Supplementary file 1 — Supplementary material 1 (PDF 1295 KB) [file 382_2018_4555_MOESM1_ESM.pdf]

Supplementary Information

The Little Ice Age signature in a 700-year high-resolution chironomid record of summer temperatures in the Central Eastern Alps

Elena A. Ilyashuk[1] • Oliver Heiri[2] • Boris P. Ilyashuk[1,3] • Karin A. Koinig[1,3] • Roland Psenner[1,3]

[1] Institute of Ecology, University of Innsbruck, Innsbruck, Technikerstraße 25, A-6020 Innsbruck, Austria
[2] Geoecology, Department of Environmental Sciences, University of Basel, Klingelbergstrasse 27, 4056 Basel, Switzerland
[3] Institute for Alpine Environment, Eurac Research, Viale Druso 1, 39100 Bozen/Bolzano, Italy

Corresponding author: Elena A. Ilyashuk, e-mail: elena.ilyashuk@uibk.ac.at, tel.: +43 (0)512 507 51735

Below we provide additional information and figures to support and extend the results of the main text.

SI 1. Sediment dating and age-depth modeling

The chronology for the MUT sediment sequence was constrained by sixteen ^{210}Pb activity determinations (down to 6.7 cm depth) and three accelerator mass spectrometry (AMS) radiocarbon dates derived from terrestrial plant macrofossils for the deeper section of the core (Ilyashuk et al. 2015). Lead-210 activity was analyzed by extracting the grand-daughter ^{210}Po and counting it in an alpha spectrometer (Flynn, 1968) in the University of Gdańsk, Poland. The ^{210}Pb-based ages of recent sediments were calculated by applying the constant rate of supply (CRS) model to the unsupported ^{210}Pb inventory (Appleby and Oldfield 1978). AMS radiocarbon dating was carried out at the Poznan Radiocarbon Laboratory (Poland) and the Beta Analytic Radiocarbon Dating Laboratory (Miami, FL, USA). Given that large chronological uncertainties can arise under translation of ^{14}C ages into calendar years if ^{14}C dates are calibrated individually (Blaauw et al. 2011), all dates were calibrated simultaneously, taking into account their stratigraphic order. Stratigraphically constrained ^{14}C calibration was performed on-line using the OxCal Bayesian software (Bronk Ramsey 2009, 2010) by applying the Northern Hemisphere terrestrial IntCal13 calibration curve (Reimer et al. 2013). The age-depth relationships (Fig. S1) were established by means of Poisson mediated deposition model (*P*

Sequence) that supposes the essentially random nature of the deposition process (Bronk Ramsey 2008). A reasonable value for the *k* parameter, which is defined by the number of the deposition events per unit depth, was chosen to be of 5, taking into account fine-grained sediment deposition in the lake and fine sampling resolution (0.22-cm slicing) of the sequence. Ages were interpolated to each sample depth based a second-order polynomial regression between median values of posterior probabilities at each chronological tie point, assuming that polynomial fitting will result in a smoother age-depth curve, avoid large shifts in modeled sediment accumulation, compared to linear interpolation, and thereby provide a better approximation of the true sediment accumulation history in the lake. As with all age-depth models, each interpolated value represents one of a range of possible values for its depth and our interpretations must be considered within this context. According to the age-depth model, an average temporal resolution is ca. 4.8 years per sample in the sediment sequence. Every sample was analyzed at the top 11.5 cm of the core (resulting in the temporal resolution of the core interval of ~4–5 years), and every other one at the lower part (resulting in the temporal resolution of the interval of ~10 years).

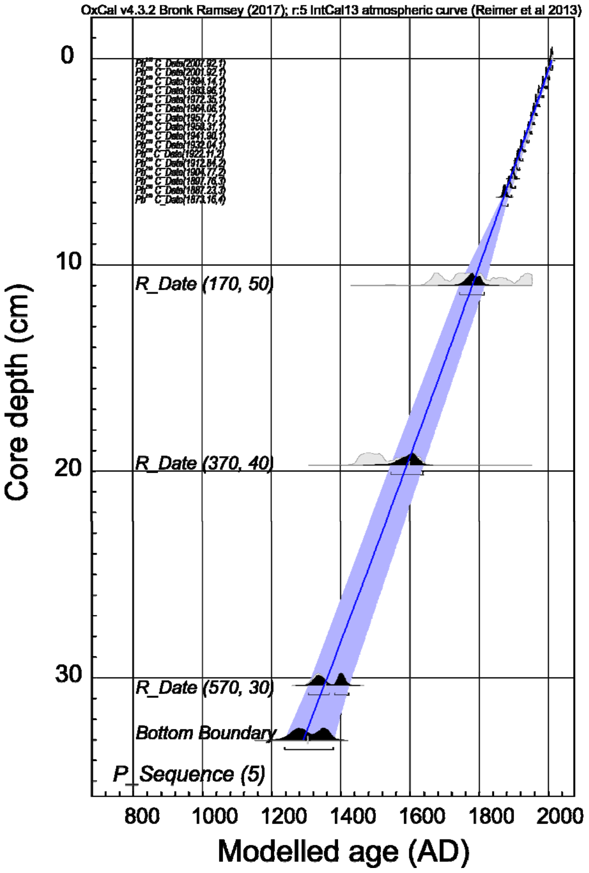

Fig. S1 The Bayesian age-depth model (*P Sequence*, k = 5) for the MUT sediment drawn through the ^{210}Pb-CRS ages and posterior distributions of calibrated ^{14}C dates. The uncertainty envelope (in blue) represents the 95.4% confidence intervals of the age model. The distributions of individually calibrated dates are shown as grey histograms. The complete age-depth curve (thick line) employed in the present study is constructed by a second-order polynomial fit between median points of the modeled ages

SI 2. Chironomid analysis

Subfossil chironomid analysis was performed on 100 sediment samples of 0.22 cm thickness, which were processed following the standard procedures for subfossil midges given in Walker (2013). Subsamples were deflocculated in 5% KOH for 30 minutes and then sieved with a 100-µm mesh. Chironomid larval head capsules were hand-picked out from the remnant in a Bogorov counting tray under a stereomicroscope at 20–40× magnification and dehydrated in 100% ethanol. Afterwards head capsules were permanently mounted ventral side up on microscope slides in Euparal® (Carl Roth Gmbh, Karlsruhe, Germany) mounting medium for identification. Chironomids were identified under a compound microscope at 200–400× magnification. Chironomid taxonomy followed Brooks et al. (2007) and Andersen et al. (2013). A minimum of 100 chironomid head capsules were counted and identified in each sample. The chironomid stratigraphy for MUT showing the relative abundance (%) of all taxa present in the lake during the past 700 years and established zonation is given in the Fig. S2.

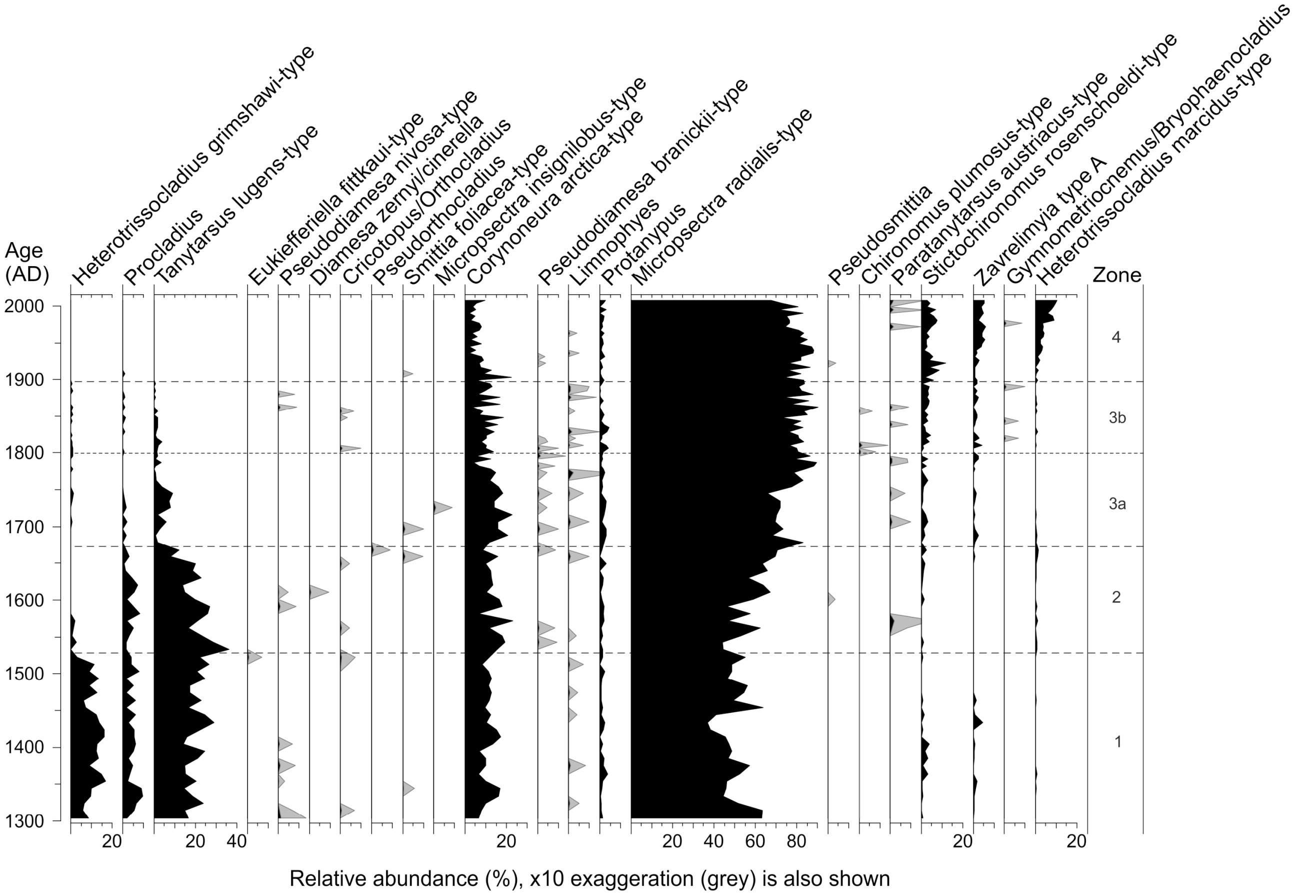

Fig. S2

Fig. S2 Chironomid diagram for MUT showing the relative abundance (%) of all taxa present in the lake during the past 700 years. Grey shading shows where x10 exaggeration has been used to improve the legibility of less abundant taxa

SI 3. Segmented regression analysis

Providing a statistical basis for spotting trends in the chironomid-inferred reconstructed temperature record, breakpoint years, with confidence intervals (CIs), and separated periods of significant trend change were determined by applying a segmented regression approach with the SegReg software (Oosterbaan 2011). In the 700-year long record, the analysis identified a significant breakpoint at AD 1783 (95% CI [1754, 1811]) (Fig. S3a). Taking into account the CI of the point and chronological uncertainties of the record, the year AD 1800 is further used as a trend changing point to estimate a multi-centennial (AD 1300–1800) cooling trend in the record (Fig. S3b). A separate segmented regression for the subsequent time interval (AD 1800–2010) revealed a breakpoint at AD 1894 (95% CI [1850, 1938]), where the horizontal stretch (no trend) is followed by a sloping line with a significant regression coefficient (Fig. S3c).

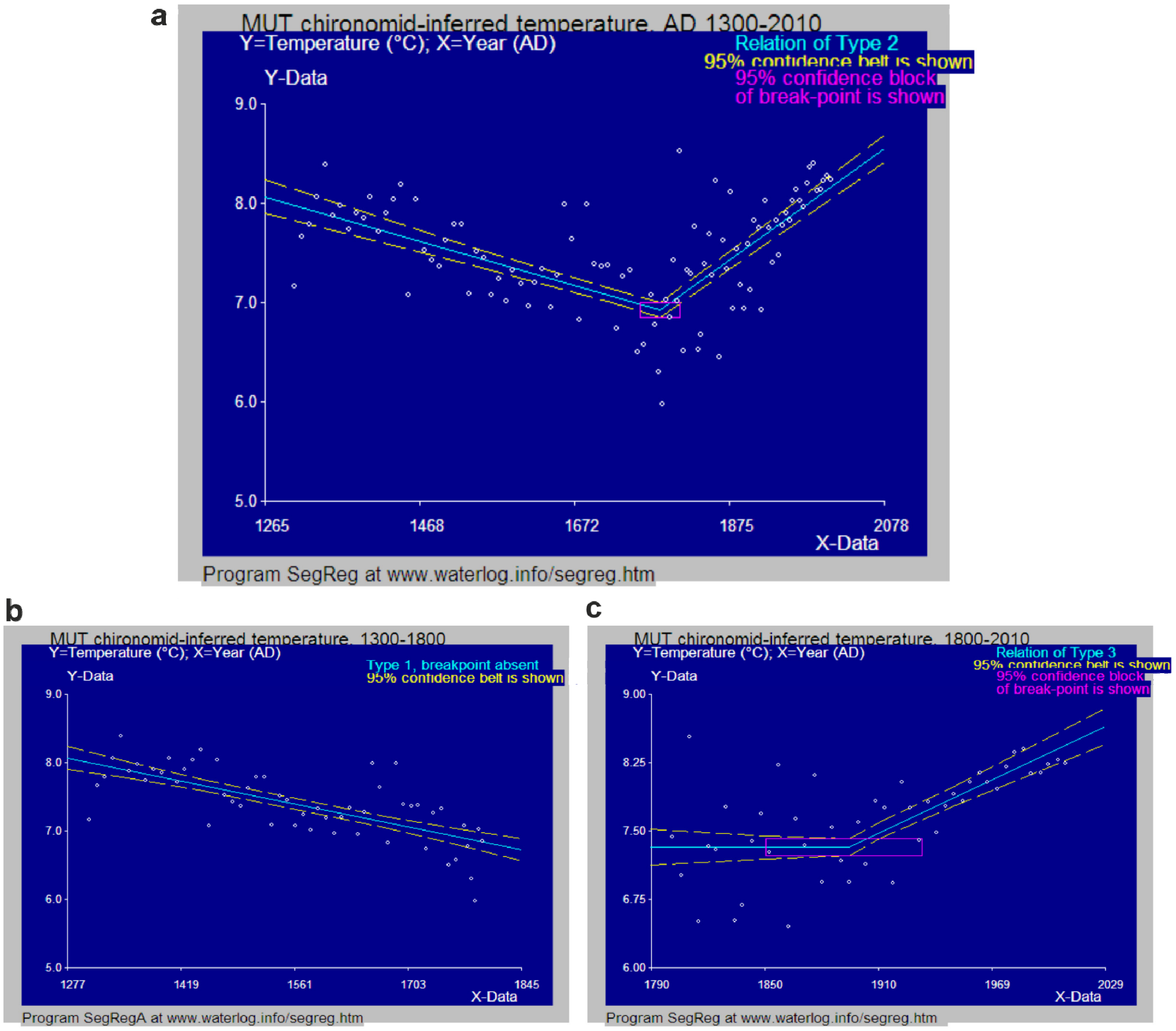

Fig. S3 (a) Segmented linear model fitted to the MUT chironomid-inferred temperature record for the time period AD 1300–2010 with a breakpoint near AD 1800. Separate segmented regression fitting of the record for **(b)** the time interval AD 1300–1800 with no significant breakpoints and **(c)** the period AD 1800–2010 with a breakpoint in the early AD 1890s

References

Andersen T, Ekrem T, Cranston PS (eds) (2013) Chironomidae of the Holarctic Region. Keys and diagnoses – Larvae. Insect Systematics and Evolution Supplement 66:1–571

Appleby PG, Oldfield F (1978) The calculation of ^{210}Pb dates assuming a constant rate of supply of unsupported ^{210}Pb to the sediment. Catena 5:1–8

Blaauw M, van Geel B, Kristen I, Plessen B et al. (2011) High-resolution 14C dating of a 25,000-year lake-sediment record from equatorial East Africa. Quat Sci Rev 30:3043–3059

Bronk Ramsey C (2008) Deposition models for chronological records. Quat Sci Rev 27:42–60

Bronk Ramsey C (2009) Bayesian analysis of radiocarbon dates. Radiocarbon 51:337–360

Bronk Ramsey C (2010) OxCal 4.1 Manual

Brooks SJ, Langdon PG, Heiri O (2007) The Identification and Use of Palaearctic Chironomidae Larvae in Palaeoecology. Quaternary Research Association, London

Flynn WW (1968) The determination of low levels of polonium-210 in environmental samples. Anal Chim Acta 43:221–227

Ilyashuk EA, Ilyashuk BP, Tylmann W, Koinig KA, Psenner R (2015) Biodiversity dynamics of chironomid midges in high-altitude lakes of the Alps over the past two millennia. Insect Conserv Divers 8:547–561

Oosterbaan RJ (2011) SegReg: segmented linear regression with breakpoint and confidence intervals. Available online: https://www.waterlog.info/segreg.htm

Reimer PJ, Bard E, Bayliss A et al. (2013) IntCal13 and Marine13 radiocarbon age calibration curves 0–50,000 years cal BP. Radiocarbon 55:1869–1887

Walker IR (2013) Chironomid overview. In: Elias SA, Mock CJ (eds) Encyclopedia of Quaternary Sciences, 2nd edn. Elsevier, London, pp 355–360
